# Supplementary figures and images for: Spirituality as a protective factor for chronic and acute anxiety in Brazilian healthcare workers during the COVID-19 outbreak
Source: PLoS One. 2022 May 3;17(5):e0267556. doi: 10.1371/journal.pone.0267556 (PMC9064089; doi:10.1371/journal.pone.0267556)

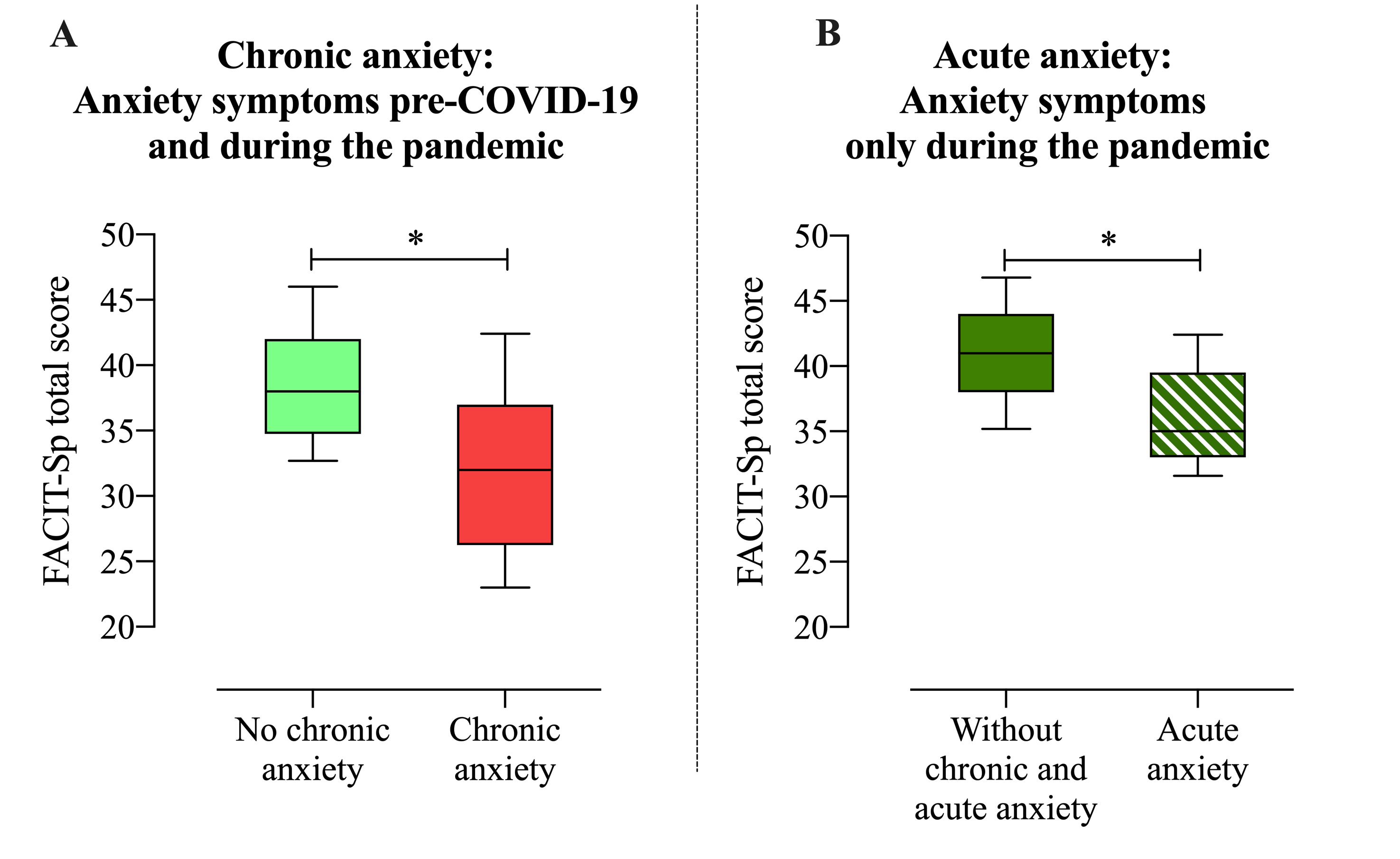

Supplement: S1 Fig — (A) FACIT-Sp total score is statistically significantly higher in the no chronic anxiety group compared to the chronic anxiety group (*p<0.001), indicating a lower spirituality among the subjects experiencing anxiety that started pre-COVID-19 and during the pandemic (B) The boxplot indicates that the FACIT-Sp total score is also statistically significantly higher in the group without chronic and acute anxiety (*p<0.001) as compared to the acute anxiety group. (TIFF) [file pone.0267556.s001.tiff]

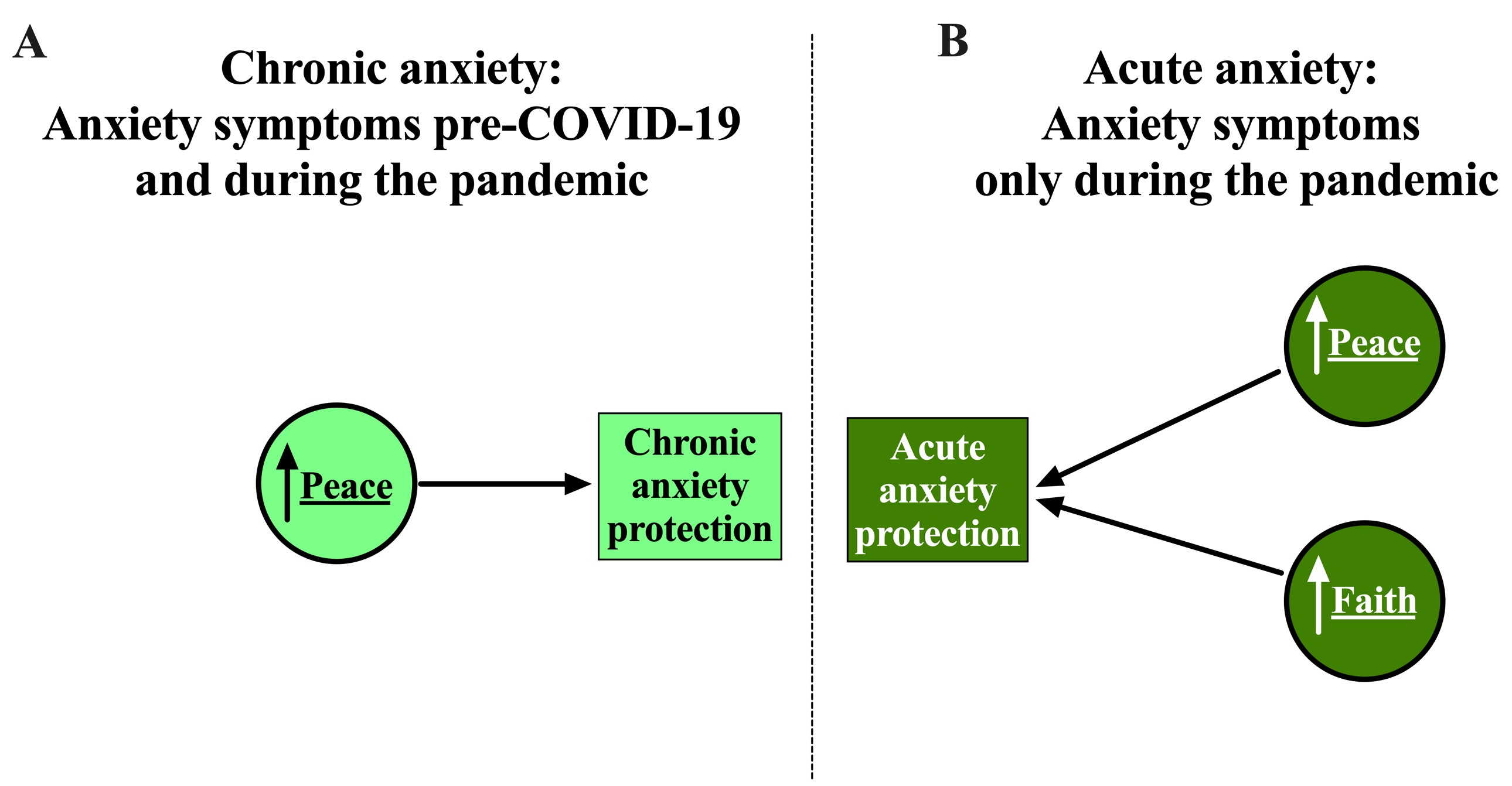

Supplement: S2 Fig — (A) The peace was the only spirituality dimension protective of chronic anxiety (B) Peace and faith spirituality dimensions are protective factors against acute anxiety during the pandemic. (TIFF) [file pone.0267556.s002.tiff]
