# Supplementary material for: Spirituality as a protective factor for chronic and acute anxiety in Brazilian healthcare workers during the COVID-19 outbreak
Source: PLoS One. 2022 May 3;17(5):e0267556. doi: 10.1371/journal.pone.0267556 (PMC9064089; doi:10.1371/journal.pone.0267556)
Supplement: Attachment — Submitted filename: PONE-D-21-18892_reviewer..docx [file pone.0267556.s003.docx]

PONE-D-21-18892

I am pleased to read and review manuscript ID PONE-D-21-18892 entitled " Spirituality as a protective factor for anxiety symptoms in healthcare workers during the COVID-19 outbreak". The study is interesting; however, I consider that specific questions need to be addressed to improve its presentation, which I mention above:

1. The abstract contains sufficient background to understand the problem under investigation. However, please focus and provide the gap statements briefly in your abstract.
2. Line 34, Page 2. Age and sex are not main variable, suggest not include in the abstract.
3. Please provide your result with related the spirituality as a protective factor for anxiety symptoms in result section-abstract.
4. Please consistent to use the word “before and during the pandemic” or “non-pandemic and pandemic periods”
5. Concerning the introduction section, more specific information is needed to provide the novelty, including what and why this study is important among Brazilian healthcare workers (HCWs) with specific spiritual and anxiety symptoms.
6. Please more clearly about the sentences in line 69-70 “However, the effects of each spirituality dimension on the AS of HCWs have not been studied during the COVID-19 outbreak”. Is it valid or true based on the previous study?
7. Line 71-15. The present study aimed to investigate AS predictors in HCWs, including spirituality, considering two different starting times for the AS. First, we assessed AS starting before the COVID-19 outbreak (first objective). Thereafter, we studied the predictors for AS conversion during the pandemic (second objective.) Finally, for each period, we analyzed which spirituality dimensions would predict AS (third objective).

Considering your objective please provide your results based on the objectives in the abstract section, especially the third objective?

8. In line 77-78, Page 3. This study was carried out in HCWs from 12th May until 9th July 2020 at a reference University Hospital for COVID-19 in Rio de Janeiro, Brazil. Based on the time. Please more detail about the time (starting before the COVID-19 outbreak-first objective; and during the pandemic-second objective) or pandemic and pandemic period
9. Line 78-80. Exclusion criteria: age below 20 years or above 60 years, previous or current neurological disorders, uncontrolled clinical conditions, and taking antidepressant, anxiolytic, and antipsychotic medications.
a. Please measure your criteria exclusion age bellow 20 years-related the HCWs or general population?
b. How about the authors clarified or measure the validity of previous or current neurological disorders, uncontrolled clinical conditions, and taking antidepressant, anxiolytic, and antipsychotic medications?
10. Please provide more detail about the validity and reliability FACIT-Sp among Brazilian as well as provide your validity and reliability (based on your results) each domain because the author used each domain
11. Line 95-97. “Normally distributed continuous variables (age and FACIT-Sp total score) are presented as ~~the~~ means and standard deviations and were evaluated using an independent t-tests. As well as “The associations are presented as odds ratios (OR) and 95% confidence intervals (CI 95%)”. Please provide the result, could be easier to use the table related the all objective (before, during the pandemic and each period).
12. The methods: needs to be more complete, I suggest the study design, the specification of the instruments with the references with the best-fitting model.
13. Please provide references related to the minimum sample size and the kind of assessment of sample size method
14. Methods in this manuscript are generally too vague. They need to be more descriptive and clearer in defining inclusion the subjects.
15. Line 111, please provide the detail of ethical approval ethic of this study.
16. In the discussion section, please describe the reason or mechanism of spirituality might protective anxiety symptoms.
17. Please provide data value of sex, age, and religious affiliation based on the objective
18. Line 160. “Sex, age, and religious affiliation were not included in the logistic regression equation because we have applied the forward analysis”. Please provide more detail about the reason and previous studies to similar or contrast about the findings among HCWs (don’t general population).
19. Should include potential other confounding variables that could be related to spiritual and anxiety symptoms. The data did not represent the entire population of health-care workers and the services in which they worked (intensive care, primary care. . .), also, did not include other variables such as whether the participants had had any personal experience of loss or illness due to COVID in their family or friends, and, as a result, the findings cannot be used to make useful generalizations regarding health-care workers as a whole, or to determine specific variables’ correlations with specific groups of health-care workers. A larger sample of health-care workers recruited from various areas in Brazil is needed to verify the results.
