# Supplementary material for: Spirituality as a protective factor for chronic and acute anxiety in Brazilian healthcare workers during the COVID-19 outbreak
Source: PLoS One. 2022 May 3;17(5):e0267556. doi: 10.1371/journal.pone.0267556 (PMC9064089; doi:10.1371/journal.pone.0267556)
Supplement: Attachment — Submitted filename: Response to Reviewers .docx [file pone.0267556.s004.docx]

**Dear Emily Chenette, PhD - Editor-in-Chief – PLOS ONE**

**Dear Rosemary Frey, PhD - Academic Editor – PLOS ONE**

We are glad to know that, based on the advice received, our manuscript entitled “Spirituality as a protective factor for anxiety symptoms in healthcare workers during the COVID-19 outbreak " has merit for publication. We thank the Editor and two anonymous reviewers for the time spent reviewing our manuscript and for the valuable input given to improve it.

We send a marked-up copy of the revised version (RV) that highlights changes made to the original version (“Revised Manuscript with Track Changes”). The changes can be found in red letters. The related question raised by the reviewers (reviewer 1- R#1 and reviewer 2- R#2) can be found highlighted in yellow (R#1) and green (R#2) on the right margin of the “Revised Manuscript with Track Changes”.

Please find below the detailed point-by-point responses to all the interesting and constructive questions (Q) raised by the two reviewers.

**Reviewer #1:**

***“I am pleased to read and review manuscript ID PONE-D-21-18892 entitled " Spirituality as a protective factor for anxiety symptoms in healthcare workers during the COVID-19 outbreak". The study is interesting; however, I consider that specific questions need to be addressed to improve its presentation, which I mention above:”***

We greatly appreciate R#1 for his/her meaningful comments on our manuscript. As R#1 points out, we agree that specific questions need to be addressed to improve our manuscript. So, we have answered the 19 questions raised by R#1 described below.

**Q1 R#1: *“The abstract contains sufficient background to understand the problem under investigation. However, please focus and provide the gap statements briefly in your abstract.”***

**Answer Q1 R#1:** We are glad that ***“The abstract contains sufficient background to understand the problem under investigation.”.*** We agree with the following interesting comment made by R#1:***"please focus and provide the gap statements briefly in your abstract.".*** We have included more focus and a brief gap statement in RV of the abstract. Please see line 30, page 2.

**Q2 R#1: *“Line 34, Page 2. Age and sex are not main variable, suggest not include in the abstract.”***

**Answer Q2 R#1:** We entirely agree with the point raised by R#1. We have removed the following sentence in the RV of abstract: "Age and sex are AS predictors in HCWs”.

**Q3 R#1: *‘’ Please provide your result with related the spirituality as a protective factor for anxiety symptoms in result section-abstract.”***

**Answer Q3 R#1:** We agree with this interesting comment raised by R#1. Then, we provided our result about spirituality as a protective factor for AS starting before and during the pandemic. Please, see lines 41-43 in the results section of RV abstract.

**Q4 R#1: *“Please consistent to use the word “before and during the pandemic” or “non-pandemic and pandemic periods”***

**Answer Q4 R#1:** We thoroughly agree with R#1. As the reviewer points out, we replaced ***“non-pandemic and pandemic periods”*** with ***“before and during the pandemic”*** in the RV.

**Q5 R#1: *“Concerning the introduction section, more specific information is needed to provide the novelty, including what and why this study is important among Brazilian healthcare workers (HCWs) with specific spiritual and anxiety symptoms”***

**Answer Q5 R#1:**: We thank R#1 for this thoughtful comment. In the RM introduction section, we included why our study is important among Brazilian HCWs, focusing on anxiety symptoms and potential protective factor of spirituality against these symptoms in HCWs. Please see lines 53-55, first paragraph (page 3), and lines 57-62, second paragraph (page 3).

**Q6 R#1: *“Please more clearly about the sentences in line 69-70 “However, the effects of each spirituality dimension on the AS of HCWs have not been studied during the COVID-19 outbreak”. Is it valid or true based on the previous study?”***

**Answer Q6 R#1:** To our knowledge, the effect of spirituality dimensions (peace, meaning, and faith) on AS in HWCs have not been studied in the current pandemic. Then, it is valid and true based on the literature review of previous studies. Please see lines 71-75 (pages 3 and 4).

**Q7R#1: *“Line 71-15. The present study aimed to investigate AS predictors in HCWs, including spirituality, considering two different starting times for the AS. First, we assessed AS starting before the COVID-19 outbreak (first objective). Thereafter, we studied the predictors for AS conversion during the pandemic (second objective.) Finally, for each period, we analyzed which spirituality dimensions would predict AS (third objective).***

**Considering your objective please provide your results based on the objectives in the abstract section, especially the third objective?”**

**Answer Q7 R#1:** We would like to thank R#1 for this interesting suggestion. First, we described our three objectives in the background section of the RV abstract (lines 31-34). After, we provided our results based on each objective in the results section of the revised abstract (lines 41-45).

**Q8R#1: “In line 77-78, Page 3. This study was carried out in HCWs from 12th May until 9th July 2020 at a reference University Hospital for COVID-19 in Rio de Janeiro, Brazil. Based on the time. Please more detail about the time (starting before the COVID-19 outbreak-first objective; and during the pandemic-second objective) or pandemic and pandemic period”**

**Answer Q8 R#1:** We thank the reviewer for pointing this out. Based on our study time, all eligible HCWs filled out a face-to-face questionnaire about spirituality and AS. The presence of AS was assessed in an all-or-none fashion based on a questionnaire that included a question if these symptoms had persisted for a minimum of 6 months. As the first case of COVID-19 in Brazil was reported on February 26^th^ 2020, it was possible to identify the participants who presented AS that started before this date. Then, the sample was divided into two groups according to the presence of AS that had started before the pandemic (no-AS and AS groups)- first objective. In order to assess the AS directly associated with the pandemic period, we subdivided the sample into two other groups: no-AS subjects that remained without AS (non-converted group) and subjects who converted to AS (converted group)- second objective. Please see lines 91-97, fifth paragraph (page 4).

**Q9 R#1: Line 78-80. Exclusion criteria: age below 20 years or above 60 years, previous or current neurological disorders, uncontrolled clinical conditions, and taking antidepressant, anxiolytic, and antipsychotic medications.
a. Please measure your criteria exclusion age bellow 20 years-related the HCWs or general population?**

**Answer Q9a R#1:** We do not include individuals from the general population. To make this age issue clearer, we have removed the age range from the exclusion criteria and described it in the inclusion criteria. Please see lines 83-84 in the third paragraph (pages 4).

**b. How about the authors clarified or measure the validity of previous or current neurological disorders, uncontrolled clinical conditions, and taking antidepressant, anxiolytic, and antipsychotic medications?**

**Answer Q9b R#1:** We thank R#1 for this relevant question. In addition to the face-to-face questionnaire carried out by each participant, we were able to verify these exclusion criteria through data obtained in the periodic healthy examination performed every six months by the occupational physician of our Hospital. Please see lines 139-140 in the second paragraph (page 6).

**Q10 R#1:** **Please provide more detail about the validity and reliability FACIT-Sp among Brazilian … reliability (based on your results) each domain because the author used each domain**

**Answer Q10 R#1**. In the RV, we described the validity and reliability of FACIT-Sp among Brazilian and provided our data of the FACIT-Sp and its domains. Please see lines 107-112, first paragraph (page 5).

**Q11 R#1: Line 95-97. “Normally distributed continuous variables (age and FACIT-Sp total score) are presented as the means and standard deviations and were evaluated using an independent t-tests. As well as “The associations are presented as odds ratios (OR) and 95% confidence intervals (CI 95%)”. Please provide the result, could be easier to use the table related the all objective (before, during the pandemic and each period).**

**Answer Q11 R#1:** We fully agree with R#1. We displayed our results in a table. Then, we include “Table 1” in the RV.

***Q12 R#1: “The methods: needs to be more complete …”***

**Answer Q12 R#1:** We thank the reviewer for pointing this out. All adjustments and additions of new sentences are in the “Material and Methods” section of the RV. Please see this section on pages 4-6 of the RV.

**Q13 R#1: “Please provide references related to the minimum sample size and the kind of assessment of sample size method.”**

**Answer Q13 R#1:** For the regression method it is well established that 15 subjects for each predictor is desirable. Hair et al. [Hair, Black, Babin, Anderson & Tatham. Multivariate Data Analysis, 2014, 7th Edition] have suggested a minimum of 10 subjects for predictor. As four predictors were used in our model (sex, age, religious affiliation, and the total FACIT-Sp score), at least 40 subjects (n=40) were needed. For the prediction analysis of the three spirituality dimensions, at least 30 individuals (n=30) were required to investigate whether these dimensions could predict significantly AS among HCWs.

**Q14 R#1: “Methods in this manuscript are generally too vague. They need to be more descriptive and clearer in defining inclusion the subjects.”**

**Answer Q14 R#1:** In the RV, we described in detail the inclusion of the subjects. Please see lines 83-84, second paragraph (page 4), and lines 86-89, fourth paragraph (page 4).

**Q15 R#1: *“Line 111, please provide the detail of ethical approval ethic of this study.”***

**Answer Q15 R#1:** We agree with R#1. In the RV, we provide more detail of the ethical approval of our study. Please see lines 133-139, second paragraph, page 6.

***Q16 R#1: “In the discussion section, please describe the reason or mechanism of spirituality might protective anxiety symptoms.”***

**Answer Q16 R#1:** In the revised version, we have included one more subsection [(“The effect of spirituality on AS starting before and during the pandemic (first and second objectives)]” to describe a potential reason or mechanism by which spirituality may protect against AS. Please see lines 220-235 (pages 10 and 11).

**Q17 R#1: *“Please provide data value of sex, age, and religious affiliation based on the objective”***

**Answer Q17 R#1:** We agree entirely with R#1. We included a table (“Table 1”) in the RV that we provided our data of sex, age, and religious affiliation based on the two different starting times (AS starting before and during the pandemic). Please see these variables displayed in “Table 1”.

**Q18 R#1: *“Line 160. “Sex, age, and religious affiliation were not included in the logistic regression equation because we have applied the forward analysis”. Please provide more detail about the reason and previous studies to similar or contrast about the findings among HCWs (don’t general population).”***

**Answer Q18 R#1:** We thank the reviewer for pointing this out. We agree that this sentence in line 160 of the original manuscript needs to be more descriptive. Then, we included some sentences in the RV. Previous studies during the pandemic have shown that sex and age are predictors of AS in HCWs (lines 50-51, page 3). However, it is not known that religious affiliation and spirituality are predictors of these symptoms in HCWs. Then, we used the forward stepwise regression method to find the best predictors of the AS. Our model considered sex, age, religious affiliation, and the FACIT-Sp total score to investigate whether these variables could significantly predict less AS among HCWs. Based on our approach, sex, age, and religious affiliation were not included as predictors in the logistic regression model (forward regression method). Spirituality was the only reliable predictor of AS in HCWs. Please see lines 123-126 (pages 5 and 6), and lines 202-205 (page 9).

**Q19 R#1: *“Should include potential other confounding variables that could be related to spiritual and anxiety symptoms… A larger sample of healthcare workers recruited from various areas in Brazil …”.***

**Answer Q19 R#1:** We agree with R#1. Then, we included this interesting comment in the limitations section of the RV. Please see lines 252-254 (page 11).

**Reviewer #2:**

***“It would be best if the discussion and conclusion sections could be expanded upon to allow for a more robust discussion of results and their direct implications of future practices for the well being of HCWs. The manuscript was very robust with statistical analysis that showed the implications of spirituality on AS, however lacked a next step for HCW. This would be helpful to add to the manuscript*.”**

We are appreciative of the R#2 encouragement and the opportunity to revise the manuscript. Basically, R#2 raised two interesting points.

**Q1 R# 2 “….*best if the discussion and conclusion sections could be expanded upon to allow for a more robust discussion of results and their direct implications of future practices for the well being of HCWs.”***

**Answer Q1 R#2:** We thank R#2 for this thoughtful comment. We expand the discussion and conclusion sections to describe a more robust discussion of the findings and their direct implications for future practice for the HCWs. Please see lines 196-200 in second paragraph (page 9); lines 222-235 (page 10 and 11); lines 242-250, second paragraph (page 11); lines 257-262, first paragraph (page 12); lines 263-266, second paragraph (page 12); lines 269-271, third paragraph (page 12); lines 275-278, fourth paragraph (page 12).

**Q2R# 2  *“...very robust with statistical analysis that showed the implications of spirituality on AS, however lacked a next step for HCW.”***

**Answer Q2 R#2:** We thank R#2 for this interesting comment. Please see lines 260-262, first paragraph (page 12); lines 263-266, second paragraph (page 12); lines 277-278, fourth paragraph (page 12).

The revised manuscript incorporated all the thoughtful suggestions and comments made by the two anonymous reviewers. The manuscript is considerably improved after taking into account all the interesting points raised by the reviewers.

We hope it will now merit publication in *PLOS ONE.*

Sincerely

Julio Cesar Tolentino, M.D, Ph.D.
