# Supplementary material for: Spirituality as a protective factor for chronic and acute anxiety in Brazilian healthcare workers during the COVID-19 outbreak
Source: PLoS One. 2022 May 3;17(5):e0267556. doi: 10.1371/journal.pone.0267556 (PMC9064089; doi:10.1371/journal.pone.0267556)
Supplement: Attachment — Submitted filename: Response to Reviewers.docx [file pone.0267556.s005.docx]

**Prof. Emily Chenette, Ph.D. - Editor-in-Chief – PLOS ONE**

**Prof. Rosemary Frey, Ph.D. - Academic Editor – PLOS ONE**

Dear Editors,

We are glad to know that, based on the first two anonymous reviewers, our revised manuscript entitled “Spirituality as a protective factor for chronic and acute anxiety in Brazilian healthcare workers during the COVID-19 outbreak " has merit for publication. We thank the Editors and the first two reviewers for the time spent reviewing our manuscript and for the valuable input given to improve it. The **Reviewer 1** concluded that “***The study is interesting and I suggest to publish”*** and the **Reviewer 2** said that **“*The manuscript was very robust with statistical analysis that showed the implications of spirituality on AS*”.** After this first major revision, the two Reviewers agreed that all questions were correctly addressed in the revised manuscript (RM). Meanwhile, the Editors invited a third reviewer. After receiving the comments raised by all the three reviewers, the final decision was a **minor review** that should address the **new** points raised by the third Reviewer.

The **Reviewer 3** (R#3) stated that “*This manuscript describes the relevance of spirituality/religion to anxiety symptoms among Brazilian healthcare workers (HCWs) prior to and during the CV19 pandemic****. The topic is novel, data (assessment of pre- and post-CV19 anxiety) is very novel, and the paper is worthwhile.*** *However, the manuscript would benefit from a number of changes.”.* We greatly appreciate his/her meaningful comments on our manuscript.

Please find below the detailed point-by-point responses to all the interesting questions (Q) raised by the R#3.

**Q1: “*Abstract: Description of the study methods is unclear - the authors did NOT have two starting times rather participants were assessed at a single time-point during the CV19 pandemic and asked retrospectively about pre-CV19 symptoms of anxiety. Further, the word "converted" is inappropriate and a bit odd given that authors assessed spirituality/religion; authors should rephrase that participants were divided into those with chronic anxiety (pre-CV19 and during) vs. acute anxiety (only during CV19) and this language should be used throughout the paper.”***

**Answer Q1:** We agree with the point raised by the R#3. In the abstract of the RM, we stressed that participants were assessed at a single time-point during the pandemic. Then, we divided the subjects into chronic anxiety (anxiety that started pre-COVID-19 and persisted during the pandemic) vs. acute anxiety (anxiety that started only during the pandemic). These terms (chronic and acute anxiety) were incorporated in the abstract, figures (Fig.1 and Fig. 2), table 1, legends, title, and throughout the RM.

**Q2: *“Abstract: … Reference to the specific dimensions of spirituality should be omitted from the abstract since readers are likely not yet familiar w/the measure or its sub-dimensions without having read the paper.”* Answer Q2:** As our results are based on these specific dimensions, we believe that we should keep this information in the abstract.

**Q3: “*Importantly: The study was conducted in Brazil yet this is omitted from the title and abstract.”***

**Answer Q3:** We entirely agree with this interesting comment raised by the R#3. Please see the revised title and abstract (line 35- page 2).

**Q4: *“Introduction: Justification of the study should be stronger - authors simply claim that age and gender are relevant and spirituality should be examined. Further, authors do not review existing research on spirituality/religion and anxiety, which are more complex than a simple buffering effect - see*** [***https://psycnet.apa.org/record/2020-20098-003***](https://psycnet.apa.org/record/2020-20098-003) ***for a recent review. The rationale for looking at spirituality among HCWs is also absent...”.***

**Answer Q4*:*** In the revised introduction we stressed that spirituality involves cognitive and emotional states such as beliefs, motivations, and a sense of gratitude as pointed out by **Rosmarin & Bethany (2020).** Positive cognitive or emotional aspects of spirituality have been found to be associated with less anxiety **(**[***https://psycnet.apa.org/record/2020-20098-003***](https://psycnet.apa.org/record/2020-20098-003)**)**. Please see lines 85-87 (page 3). **Moreover, we also make clear that González-Sanguino et al. (Brain Behav Immun. 2020;87: 172–176), using regression methods, have demonstrated the importance of spirituality as the main protector against appearance of anxiety.** Please see lines 88-89 (page 3).

In the RM introduction, we fully extended the rationale for looking at spirituality among HCWs. Please see the introduction of the revised version (lines 90-93- page 3).

**Q5: *“Introduction … The context of the study - Brazil - should be mentioned as a justification, since spirituality/religion is commonplace within Brazilian culture.”***

**Answer Q5:** We thank the reviewer for pointing this out. Please see lines 99-111, pages 3-4.

**Q*6…The measure need not be introduced in the introduction…”***

**Answer Q6:** We still believe that a brief description of the FACIT should be present in the introduction because we extensively used the spirituality dimensions derived from this questionnaire.

**Q7: *“Introduction: … Relevance of CV19 to both anxiety and spirituality should be explained: Why might spirituality be more (or less?) relevant to HCWs with anxiety that preceded CV19 vs. those experiencing acute anxiety during the pandemic without a history of anxiety? Hypotheses should be provided along these lines - e.g., Many people increase spirituality in times of distress and those without a history of anxiety may benefit less from spirituality, Alternatively, those with chronic anxiety may stand to benefit the most from spirituality. The authors need to justify their methods and approach more clearly.”***

**Answer Q7:** In the RM we make clear that we did not perform any measure of a putative increase of spirituality. We did not assess spirituality before the pandemic. Although many people may increase spirituality in times of distress, this interesting question entails a new project. However, we pointed out that healthcare workers are under stressful conditions during the pandemic (lines 124-126, page 4).

As there is an increase in the number of subjects with anxiety in times of distress, it is conceivable that higher basal protection should be needed to overcome the demands of a new stressful situation. Therefore, some people without anxiety symptoms preceding the COVID-19 might not be entirely protected during the pandemic. We hypothesized that subjects without pre-pandemic anxiety with higher spirituality at the baseline should be able to overcome the stressful time during the pandemic. Therefore, given the potential protection of greater spirituality against anxiety [12–16], we hypothesized that those experiencing acute anxiety during the pandemic would exhibit lower basal spirituality, as compared to those who remained without anxiety before and during the pandemic.

In addition, differences in spirituality scores would reflect differences in specific spirituality dimensions. As personal faith can increase psychological resilience [45,46], we hypothesized that faith would reach the highest score in those without chronic and acute anxiety. In addition, as faith is associated with better coping in stressful times [47–49], we also hypothesized that higher personal faith could be a potential predictor of less acute anxiety during the pandemic. Please see lines 131-134, second paragraph (page 4).

We would like to stress that we have used the definitions according to the R#3 suggestions *(“…****authors should rephrase that participants were divided into those with chronic anxiety (pre-CV19 and during) vs. acute anxiety (only during CV19) and this language should be used throughout the paper.”)***

**Q8: *“Methods: Sampling method is inadequately described; was this a convenience sample? How were participants recruited? How many refused to participate? Were they compensated?”***

**Answer Q8:** In the Methods section of the RM, we answered these interesting questions raised by the R#3. Please see lines 157-158, third paragraph (page 5), lines 227-228, second paragraph (page 8), and lines 238-239, third paragraph (page 8).

**Q9: *“… Why did the authors only select participants without AS before the pandemic??”***

***As is, the authors examined the relevance of spirituality to anxiety, among participants with no significant anxiety - what is the relevance of such an approach?!”***

**Answer Q9:** Thanks for your comment, but we aimed to investigate whether spirituality would predict anxiety that started before the pandemic and persisted during the pandemic (chronic anxiety) and anxiety that started only during the pandemic (acute anxiety).

We select participants without anxiety symptoms before the pandemic in order to investigate the predictors of acute anxiety during the pandemic- Please see lines 172-176, first paragraph (page 6).

Again, we did not measure spirituality before the pandemic because it was not included in our objectives.

**Q10: *“Similarly they excluded individuals using psychotropic medications - why would they do this considering that the main variable under study is clinical?”***

**Answer Q10:** We excluded individuals using psychotropic medications to avoid a potential protective effect of these drugs on the development of anxiety symptoms, especially in those who started these symptoms only during the pandemic (acute anxiety). Furthermore, it should be mentioned that these medications could also be used to treat some clinical diseases.

**Q11:** ***“Another concern pertains to the measure of spirituality. As Koenig and others have explained, the FACIT assesses multiple dimensions of "spirituality" and the peace sub-scale is more akin to an assessment of mental health than spiritual/religious life. Thus******, a significant negative relationship between "peace" and anxiety is not very meaningful. By contrast, the "faith" subscale would yield more interesting results. Again, I encourage the authors to examine these variables among individuals with chronic vs. acute anxiety within their sample.”***

**Answer Q11:** We thank the R#3 for pointing this out. Previous studies on the peace domain derived from the FACIT-SP have been shown that this dimension reflects an affective component of spirituality [35,36,41,42]. In addition, the peace dimension correlates with mental health [11,43,44], and some researchers have reported a significant negative relationship between "peace" and anxiety [11,43]. Furthermore, we agree with the R#3 that the findings of the present study about the faith spirituality dimension are more interesting. Indeed, we found that the faith dimension emerged together with peace to confer protection on acute anxiety starting only during the COVID-19 outbreak.

As suggest by R#3 we examined these variables (peace and faith spirituality dimensions) in the fourth objective of this study.

**Q12*: “Results and Discussion ….”***

**Answer Q12:** In the RM we made the changes based on the interesting comments raised by the R#3. In addition, we have included one more subsection in the Results and Discussion sections regarding the fourth objective. Please see the Results and Discussion sections in the revised version.

The revised manuscript incorporated all the thoughtful suggestions and comments made by the three anonymous reviewers. After responding to the new points raised by the R#3, we hope it will now merit publication in *PLOS ONE.*

Sincerely

Julio Cesar Tolentino, M.D, Ph.D.
